# Atomic basis for therapeutic activation of neuronal potassium channels

**DOI:** 10.1038/ncomms9116

**Published:** 2015-09-03

**Authors:** Robin Y. Kim, Michael C. Yau, Jason D. Galpin, Guiscard Seebohm, Christopher A. Ahern, Stephan A. Pless, Harley T. Kurata

**Affiliations:** 1 Department of Anesthesiology, Pharmacology, and Therapeutics, University of British Columbia, 2176 Health Sciences Mall, Vancouver, British Columbia, Canada V6T 1Z3; 2 Department of Molecular Physiology and Biophysics, University of Iowa, 285 Newton Road, Iowa City, Iowa 52242, USA; 3 Department of Cardiovascular Medicine, University Hospital Münster, Albert-Schweitzer-Campus 1 (Gebäude D3), D-48149 Münster, Germany; 4 Department of Drug Design and Pharmacology (Center for Biopharmaceuticals), University of Copenhagen, Jagtvej 160, DK-2100 Copenhagen, Denmark

## Abstract

Retigabine is a recently approved anticonvulsant that acts by potentiating neuronal M-current generated by KCNQ2–5 channels, interacting with a conserved Trp residue in the channel pore domain. Using unnatural amino-acid mutagenesis, we subtly altered the properties of this Trp to reveal specific chemical interactions required for retigabine action. Introduction of a non-natural isosteric H-bond-deficient Trp analogue abolishes channel potentiation, indicating that retigabine effects rely strongly on formation of a H-bond with the conserved pore Trp. Supporting this model, substitution with fluorinated Trp analogues, with increased H-bonding propensity, strengthens retigabine potency. In addition, potency of numerous retigabine analogues correlates with the negative electrostatic surface potential of a carbonyl/carbamate oxygen atom present in most KCNQ activators. These findings functionally pinpoint an atomic-scale interaction essential for effects of retigabine and provide stringent constraints that may guide rational improvement of the emerging drug class of KCNQ channel activators.

## Abstract (web summary)

The antiepileptic drug retigabine potentiates neuronal KCNQ potassium channels. Here, the authors use a combination of unnatural amino acid mutagenesis and electrophysiology to show that retigabine acts by hydrogen bonding with a tryptophan indole nitrogen in the channel pore.

## Graphical abstract

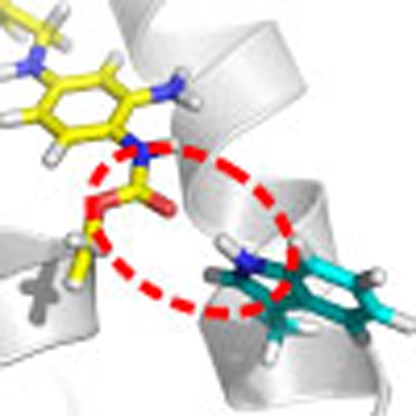

Epilepsy is a common, heterogeneous and often debilitating disorder, affecting ∼1% of the global population. Current clinical management of patients suffering from epilepsy is primarily via medication, although it is generally accepted that ∼30% of newly diagnosed epilepsy patients will be resistant to common anticonvulsant drugs^1^,^2^. Moreover, such resistance is correlated to seizure type—for example, it has been reported that 60% of patients suffering from focal epilepsy will develop and retain resistance to medications^3^. These shortcomings have motivated the search for new antiepileptic drugs, non-conventional treatments for epilepsy (such as the ketogenic diet) and the identification and ongoing validation of new drug targets^4^,^5^,^6^,^7^.

Pharmacotherapy in epilepsy has been primarily directed towards a small subset of ion channel targets^8^. Overall, the vast majority of drugs developed as anticonvulsants are targeted towards voltage-gated Na^+^ channels or GABA-mediated neurotransmission. However, several recently developed drugs have begun to expand the diversity of molecular targets for epilepsy. A good example is the ‘first-in-class’ Kv channel opener retigabine, presently approved for use as an adjunct therapy to antiepileptic drugs for partial/focal seizures^9^. Among all the antiepileptic drugs in use, retigabine has a unique mechanism—it is the only compound approved for human use that acts by activating/opening Kv channels. The resulting suppression of electrical excitation likely underlies its anticonvulsant and analgesic properties^10^,^11^. Numerous studies have demonstrated that this drug class is effective and well tolerated over a wide therapeutic window, with comparable effectiveness to many classic anticonvulsants in animal models^7^,^12^,^13^. However, despite the therapeutic potential for management of epilepsy and disorders involving membrane excitability, there remains a paucity of molecular understanding of the binding mode(s) and mechanism of action of this new drug class.

Retigabine acts primarily by opening KCNQ2–5 (Kv7.2–7.5) channels^11^,^14^,^15^. In the nervous system, KCNQ2–5 channels assemble as heteromers to generate the ‘M-current’, a subthreshold voltage-gated K^+^ conductance that is influenced by muscarinic receptor signalling (primarily via PIP_2_) and regulates neuronal excitability^14^,^16^. Retigabine activates these channels by shifting their voltage dependence to hyperpolarized membrane potentials, effectively causing them to open at voltages where they would normally adopt a closed, non-conducting conformation^17^,^18^. A conserved tryptophan residue in the pore-forming S5 helix of retigabine-sensitive KCNQ channels (KCNQ2 Trp236, KCNQ3 Trp265) is known to be essential for retigabine effects and is absent in the retigabine-resistant cardiac isoform KCNQ1 (refs 19, 20). The identification of retigabine as a potent Kv channel opener has led to numerous compound library screens to generate novel analogues with altered subunit specificity and effectiveness^9^,^21^,^22^,^23^,^24^,^25^. However, the molecular details that underlie retigabine–KCNQ channel interactions have remained elusive because of the inherent lack of resolution of conventional site-directed mutagenesis techniques.

We have used unnatural amino-acid mutagenesis to subtly rearrange atoms and electrons of the Trp side chain in the retigabine binding site. Our findings demonstrate that repositioning of the indole nitrogen atom of KCNQ3 Trp265 completely abolishes retigabine effects, suggesting that retigabine interaction requires the formation of a H-bond with Trp265. The importance of this H-bond interaction is further illustrated by introduction of fluorinated Trp analogues (with increased H-bonding propensity) at KCNQ3 Trp265, causing increased drug potency. Using multiple retigabine analogues, we identify an electrostatic fingerprint around a H-bond acceptor that correlates with drug potency. These experimental constraints pinpoint specific atoms and chemical forces involved in retigabine interactions, and highlight approaches that may be used to guide rational improvement of existing drugs.

## Results

### Retigabine modulation of KCNQ channels through an S5 Trp

**Figure 1:**
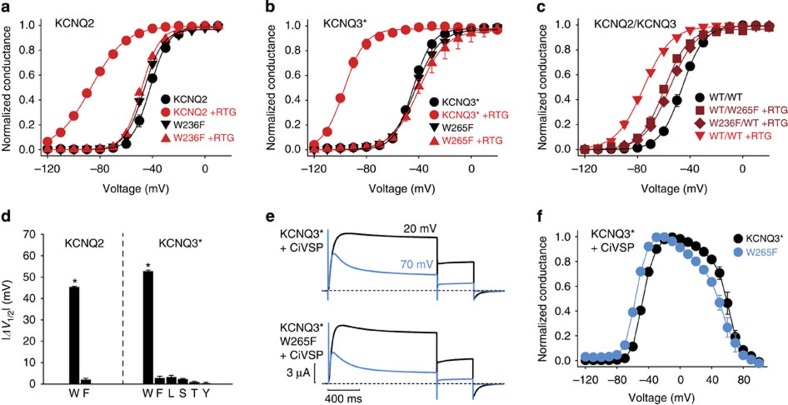
Multiple retigabine molecules modulate KCNQ2 and KCNQ3 channel subunits via an S5 Trp side chain. (**a**,**b**) Conductance–voltage relationships for (**a**) KCNQ2 (*n*=3) and KCNQ2[Trp236Phe] (*n*=6), and (**b**) KCNQ3* (*n*=5) and KCNQ3*[Trp265Phe] (*n*=3) homomeric channels along with indicated mutants (retigabine concentration of 100 μM). (**c**) Conductance–voltage relationships for heteromeric combinations of KCNQ2 and KCNQ3 (1:1 ratio of injected mRNA, with or without Trp→Phe mutations as indicated, *n*=5 for each combination), used to generate channels with reduced numbers of retigabine binding sites. (**d**) Summary of *V*_1/2_ shifts in saturating 100 μM retigabine for mutations of KCNQ2 Trp236 and KCNQ3 Trp265 as indicated (*P<0.05 in a paired Students *t*-test comparing control versus 100 μM retigabine in each experimental oocyte, *n*=3–6 per mutant). Only a Trp at either position is sufficient for retigabine sensitivity. (**e**) Exemplar currents of KCNQ3* and KCNQ3*[Trp265Phe] mutant coexpressed with CiVSP, illustrating that the Trp side chain responsible for retigabine sensitivity is not required for PIP2 sensitivity. (**f**) Summary data of tail current magnitude (−20 mV) after prepulses to a range of voltages, in oocytes expressing KCNQ3* (*n*=5) or KCNQ3*[Trp265Phe] (*n*=5) channels, along with CiVSP. In all panels, error bars represent s.e.m.

Retigabine strongly affects voltage-dependent gating of KCNQ2 and KCNQ3* channels (Fig. 1a,b) by generating a substantial hyperpolarizing shift of the *V*_1/2_ of activation by ∼40–60 mV (KCNQ3* refers to KCNQ3[Ala315Thr]—see Methods). These effects have also been demonstrated in KCNQ4 and KCNQ5, but are absent in KCNQ1 because of the absence of an essential Trp residue (Trp236 in KCNQ2, Fig. 1a; Trp 265 in KCNQ3, Fig. 1b: Leu246 in KCNQ1)^14^,^26^. This Trp side chain lies in the pore-forming S5 transmembrane segment, near the intracellular voltage-operated gate of the channel. As KCNQ2–5 subunits generally assemble as heteromers in the central nervous system^14^,^16^, we tested the effects of retigabine in oocytes co-injected with KCNQ2 and KCNQ3 and observed large shifts of activation to more negative voltages, although not quite as large as with KCNQ2 or KCNQ3 alone (Fig. 1c). Some KCNQ channel activator molecules also cause a marked increase in peak current; however, the effects of retigabine on KCNQ3* channels are quite modest (rarely greater than a 15% increase in peak current), and throughout this study we have focused on the large gating shifts observed in these channels.

Heteromeric KCNQ2/3 channels were used to alter the number of available retigabine binding sites, by co-injecting KCNQ2[Trp236Phe] with wild-type (WT) KCNQ3, or KCNQ3[Trp265Phe] with WT KCNQ2. Assuming 1:1 stoichiometry of KCNQ2/3 assembly, both conditions are estimated to eliminate roughly half of the retigabine binding sites. Consistent with this, we observed intermediate effects of retigabine with fewer available binding sites, indicating that multiple molecules of the drug may be required to generate the full effect (Fig. 1c). A surprising, currently unexplained observation, was that the retigabine-mediated gating shift in KCNQ2/3 heteromers (∼−40 mV) was noticeably smaller than the shift in KCNQ2 or KCNQ3* homomeric channels. This likely hints that determination of the exact details of stoichiometry and cooperativity of drug binding in heteromeric channels will require alternative experimental approaches. We measured the effects of various mutations at position Trp265 (in KCNQ3*) or Trp236 (in KCNQ2) on retigabine sensitivity (Fig. 1d). In both KCNQ2 and KCNQ3* channels, only a Trp side chain present at this position was sufficient to enable a significant gating shift by retigabine, and even conservative mutations to other aromatic side chains (Phe or Tyr, Fig. 1a,b,d) cannot replicate the retigabine-mediated gating shift, consistent with previous reports^19^,^20^.

We tested whether KCNQ3 position Trp 265 plays a role in regulating channel responses to PIP_2_. Using the voltage-sensitive phosphatase ciona intestinalis voltage-sensitive phosphatase (CiVSP) to hydrolyse PIP_2_ at depolarized voltages, we pulsed oocytes through a range of prepulse voltages followed by a test pulse to −20 mV to assess channel activity after PIP_2_ depletion^27^. We observed similar channel rundown with depolarizing voltage steps in both KCNQ3* and KCNQ3*[Trp265Phe] channels, indicating that Trp265 does not significantly influence the effects of anionic phospholipids on channel function (Fig. 1e,f). Overall, these findings illustrate that KCNQ3 residue Trp265 is essential for the effects of retigabine, but does not play a significant role in regulating channel gating by voltage or PIP_2_.

### Unnatural amino-acid mutagenesis of KCNQ3 channels

**Figure 2:**
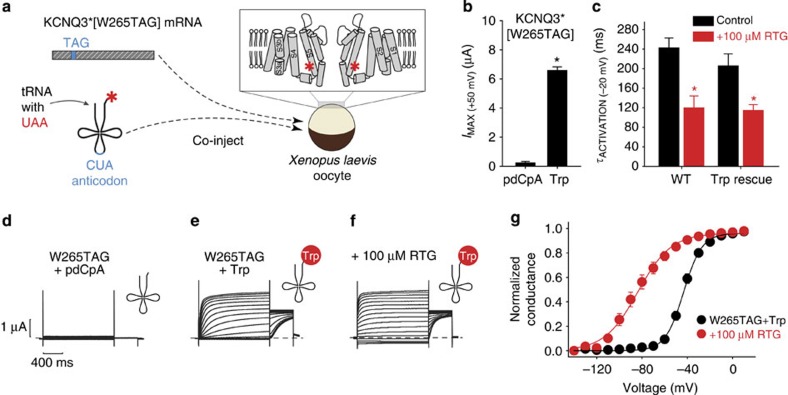
Nonsense suppression for amino-acid incorporation in KCNQ3* channels. (**a**) Schematic diagram of the nonsense suppression method, in which mRNA (with a stop codon at Trp265) and amino-acylated tRNA are co-injected into *Xenopus* oocytes. Incorporation of the unnatural amino acid enables readthrough of the stop codon and expression of functional channels. (**b**) Current magnitudes in oocytes injected with KCNQ3*[Trp265TAG] mRNA and either an unconjugated tRNA (pdCpA; *n*=5) or tRNA amino-acylated with Trp (*n*=8, *P<0.05, Student’s *t*-test). (**c**) Activation kinetics of KCNQ3* (*n*=5) and KCNQ3*[Trp265TAG] (*n*=8) channels rescued with Trp, in the presence or absence of 100 μM retigabine (*P<0.05, Student’s *t*-test). (**d**–**f**) Exemplar currents from oocytes injected with KCNQ3*[Trp265TAG] mRNA, and indicated synthetic tRNAs. (**g**) Conductance–voltage relationships for Trp-rescued KCNQ3*[Trp265TAG] (*n*=8), with retigabine response, illustrating faithful incorporation of the desired side chain at position 265. For KCNQ3* channels *V*_1/2_=−44±1 mV, *k*=7.5±0.5 mV; for Trp-rescued KCNQ3*[Trp265TAG] *V*_1/2_=−43±2 mV, *k*=7.9±0.5 mV (± indicates s.e.). Error bars throughout represent s.e.m.

To investigate the underlying mechanism of retigabine interactions with this essential Trp side chain, we employed unnatural amino-acid mutagenesis to introduce subtly altered Trp variants (Fig. 2a). With this method, a stop codon (TAG) is placed in the ion channel gene at a site of interest, and this mRNA is co-injected into *Xenopus laevis* oocytes along with a synthetic amino-acylated tRNA (carrying an unnatural amino acid) that is orthogonal to *Xenopus* tRNA synthetic pathways^28^. When the ribosomal translation machinery encounters the introduced TAG stop codon, the complementary synthetic tRNA (with a CUA anticodon) enables readthrough and incorporation of the appended amino acid into full-length ion channels (Fig. 2a)^29^.

Among the KCNQ channels we tested, KCNQ3* channels exhibited large currents when expressed as homomeric channels in *Xenopus* oocytes, with a short latency between injection and expression, and were found to be an effective model system for incorporation of unnatural amino acids. KCNQ3* is also a useful model for retigabine binding because it does not possess a second interaction site that is targeted by some other recently studied KCNQ2 activators (see Discussion)^22^,^30^, and so our results are specifically focused on the retigabine binding site formed by Trp265. Several criteria illustrate the feasibility and fidelity of the nonsense suppression approach in KCNQ3* channels. First, co-injection of KCNQ3*[Trp265TAG] with a full-length tRNA lacking a conjugated amino acid (pdCpA control) resulted in virtually no ionic currents, even several days after injection (Fig. 2b,d). However, when KCNQ3*[Trp265TAG] channels were co-injected with Trp-conjugated tRNA, robust currents with WT-like gating were observed, indicating efficient suppression of the introduced TAG stop codon and synthesis of functional channels with WT-like properties (Fig. 2b,e). Second, although there may be differences in the efficiency of incorporation of unnatural amino acids at this position, we have restricted our investigation to homomeric channels (rather than KCNQ2/KCNQ3 heteromers), and to biophysical parameters (*V*_1/2_, activation kinetics) that are normally independent of channel expression. Last, we are confident that the synthetic Trp-tRNA was incorporated in a significant fraction of channel subunits because elicited currents responded to retigabine (Fig. 2c,f,g), and only Trp appears to be sufficient for retigabine sensitivity (Fig. 1d).

### Loss of retigabine effects after removal of Trp265 H-bonding

**Figure 3:**
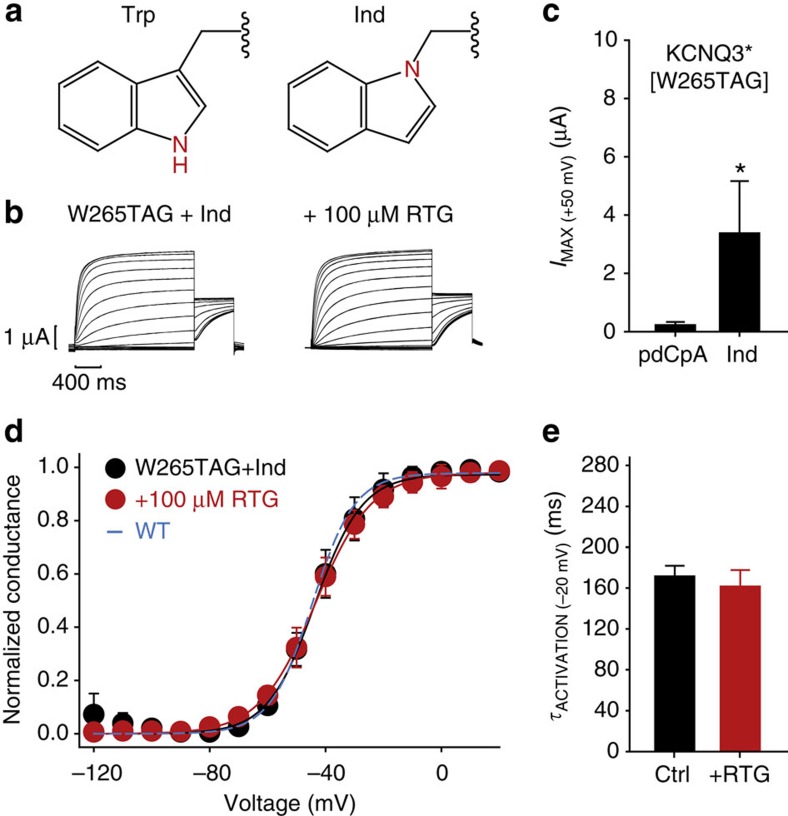
The position of the Trp 265 indole nitrogen is essential for retigabine sensitivity. (**a**) Chemical structures of Trp and Ind side chains, illustrating the subtle change in the position of the indole nitrogen atom. (**b**) Exemplar currents elicited from a Xenopus oocyte with Ind-rescued KCNQ3*[Trp265TAG] channels illustrating retigabine insensitivity. (**c**) Current magnitudes in oocytes injected with KCNQ3*[Trp265TAG] mRNA and either an unconjugated tRNA (pdCpA; *n*=5) or tRNA amino-acylated with Ind (*n*=7, *P<0.05, Student’s *t*-test). (**d**) Conductance–voltage relationships for Ind-rescued KCNQ3*[Trp265TAG], in the presence and absence of retigabine, illustrating the importance of the correct positioning of the N–H group. For KCNQ3*[Trp265Trp], *V*_1/2_=−43±2 mV, *k*=7.9±0.5 mV; for KCNQ3*[Trp265Ind], *V*_1/2_=−48±2 mV, *k*=7.3±0.6 mV (no statistical significance, ±indicates s.e.). (**e**) Activation kinetics for KCNQ3*[Trp265Ind] measured at −20 mV in the presence and absence of 100 μM retigabine (*n*=7, no statistical significance). In all panels, error bars represent s.e.m.

Next, we tested retigabine sensitivity of KCNQ3* channels synthesized with the ‘Ind’ amino acid at position 265. This unnatural amino-acid side chain ablates the hydrogen-bonding capability of Trp by shifting the position of the indole nitrogen atom (Fig. 3a)^31^, but retains the steric and chemical compositions of Trp. KCNQ3*[Trp265TAG] currents were efficiently rescued with Ind relative to the pdCpA control, yielding relatively large voltage-activated K^+^ currents that closely resembled KCNQ3* (Fig. 3b,c). Remarkably, however, this single atom alteration in each of the KCNQ3* subunits entirely abolishes retigabine activation of KCNQ3* channels (Fig. 3d,e). Notably, Trp265Ind channels displayed WT-like gating, indicating that this residue does not play a significant role in conformational stabilization of channel states in the absence of drug. However, given the potent impact of such a minor manipulation, we examined the effects of the Trp265Ind substitution on channel gating more closely, to rule out the possibility of significant perturbation of channel function. We observed no statistically significant difference in the *V*_1/2_ or slope of the voltage dependence of activation of KCNQ3* and KCNQ3*[Trp265Ind] (see Table 1 and Fig. 3d,e). Last, as described earlier, the more disruptive Trp265Phe mutation did not alter the inhibition of KCNQ3* currents by PIP_2_ depletion (mediated by CiVSP). Taken together, these observations demonstrate a highly specific effect of the Trp265Ind substitution on drug interactions (with little disruption of intrinsic voltage-dependent gating or lipid regulation), indicating that H-bond formation with Trp265 is a crucial step for retigabine action.

### Tuning the strength of the Trp265–retigabine interaction

**Figure 4:**
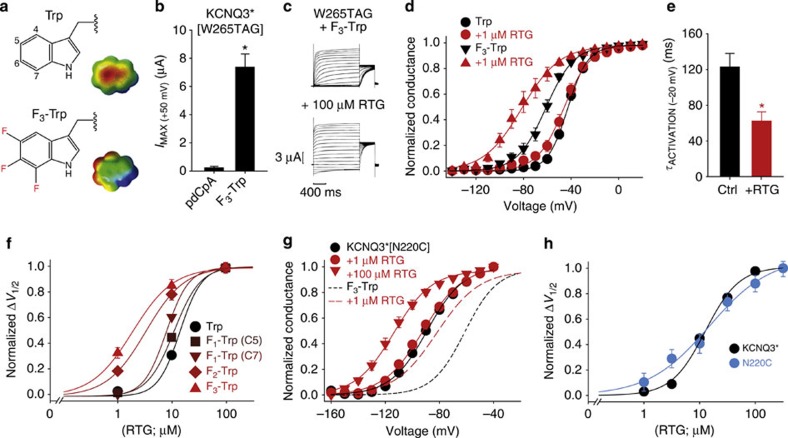
The polarity of the Trp265 indole nitrogen modulates retigabine sensitivity. (**a**) Chemical structures of Trp (with ring positions labelled) and F_3_-Trp, accompanied by colorimetric representations of electrostatic surface potentials. (**b**) Current magnitudes in oocytes injected with KCNQ3*[Trp265TAG] mRNA and either an unconjugated tRNA (pdCpA; *n*=5) or tRNA amino acylated with F_3_-Trp (*n*=12, *P<0.05, Student’s *t*-test). (**c**) Exemplar currents from F_3_-Trp-rescued KCNQ3*[Trp265TAG] channels in the presence and absence of retigabine. (**d**) Conductance–voltage relationships illustrating the effects of 1 μM retigabine on Trp-rescued (*n*=12) and F_3_-Trp-rescued KCNQ3*[Trp265TAG] (*n*=12) channels. (**e**) Activation kinetics (−20 mV) for F_3_-Trp-rescued channels (*n*=3), in the presence and absence of 100 μM retigabine (*P<0.05, Student’s *t*-test). (**f**) Concentration–response curves for retigabine effects on numerous fluoro-Trp analogues (*n*=9–12 per Trp analogue) substituted at position Trp265, illustrating enhanced retigabine potency with increased fluorination. (**g**) Conductance–voltage relationships with indicated retigabine concentrations on a KCNQ3 mutant (Asn220Cys, *n*=4) with an intrinsic hyperpolarizing shift in gating. Conductance–voltage relationships for F_3_-Trp substituted at Trp265TAG are shown for comparison. (**h**) Concentration–response curves for retigabine effects on KCNQ3* (*n*=5) and KCNQ3*[Asn220Cys] (*n*=4) channels. In all panels, error bars represent s.e.m.

To investigate the possible involvement of other modalities of Trp interactions with the drug, we also examined the consequences of fluorination of Trp265 on retigabine effects. Trp fluorination is typically used to modify the electrostatic surface potential and test for the effects of cation–pi interactions (Fig. 4a)^32^. We were unable to detect functional channels carrying the F_4_-Trp side chain (fluorines at ring positions 4,5,6 and 7, as numbered in Fig. 4a); however, robust current rescue was observed for less fluorinated derivatives such as F_3_-Trp (Fig. 4b,c). Retigabine potentiation of KCNQ3* channels was retained with F_3_-Trp substitution at Trp265 (Fig. 4c–e), a substitution that still potently diminishes the negative electrostatic potential on the face of the side chain (Fig. 4a), indicating that a cation–pi interaction of Trp265 with retigabine or some other entity (perhaps another channel residue) is not required for retigabine effects. It is noteworthy that progressive fluorination of Trp265 caused variable effects on KCNQ3*-gating properties (Table 1), with F_1_-Trp causing a modest depolarizing shift of the activation *V*_1/2_, while F_3_-Trp significantly shifted gating in the hyperpolarizing direction. This lack of an obvious trend also suggests that an endogenous cation–pi interaction involving Trp265 does not contribute significantly to the gating process.

The most interesting outcome of fluoro-Trp substitutions further highlights the role of H-bond formation by the indole N–H group. Specifically, fluorination of the Trp side chain significantly increases the polarity of the indole N–H, thereby strengthening the hydrogen-bonding propensity of the indole side chain^33^,^34^. This is illustrated by the electrostatic surface potentials for Trp and F_3_-Trp (note the more polarized electrostatic surface potential of F_3_-Trp, highlighted by the blue colour around the N–H bond, in the lower right corner as oriented in Fig. 4a). Consistent with this, retigabine potency was increased with fluoro-Trp analogues at Trp265. For instance, in KCNQ3*[Trp265TAG] channels rescued with Trp (mimicking KCNQ3* channels), 1 μM retigabine elicits very little shift in the voltage dependence of activation (Fig. 4d). However, in channels with F_3_-Trp substituted at Trp265, 1 μM retigabine elicits a much larger response (Fig. 4d). Consistent with this effect being attributable to strengthening of the formation of a hydrogen bond by Trp265, we observed a fluorination-dependent increase in retigabine potency, with progressive fluorination shifting the concentration response to lower retigabine concentrations (Fig. 4f). In addition, we observed a small but significant position-dependent effect of mono-fluorination of Trp265, in which fluorination of ring position 7 (closer to the indole N–H) increased retigabine effects slightly more than fluorination of ring position 5 (Fig. 4f). This is consistent with the fluorine substituent at position 7 having a stronger effect on the polarity of the N–H bond.

Since F_3_-Trp incorporation itself caused a hyperpolarizing shift of channel activation even in the absence of retigabine (Table 1 and Fig. 4d), a possible alternative explanation was that increased retigabine potency with F_3_-Trp at position 265 was a secondary allosteric effect of stabilization of the channel open state, rather than a direct effect on retigabine binding. Thus, as an additional control experiment, we investigated the effects of the Asn220Cys mutation on retigabine sensitivity (in the extracellular S3–S4 linker, distant from the Trp265 putative retigabine binding residue). This mutation shifts channel opening to more negative voltages, even more than F_3_-Trp substitution at position 265. In the Asn220Cys mutant, no sensitization to retigabine was observed (Fig. 4g). That is, 1 μM retigabine was unable to generate a marked gating shift, in contrast to the potent retigabine effects with F_3_-Trp substitution at Trp265 (curve fits for the F_3_-Trp substitution are included for comparison, Fig. 4g). In addition, the retigabine concentration response for Asn220Cys channels was very similar (albeit shallower) to the KCNQ3* background construct, demonstrating that the Asn220Cys mutation *per se* does not inhibit retigabine activation (Fig. 4h). These observations suggest that an indirect effect of open-state stabilization is unlikely to account for increased retigabine potency in F_3_-Trp-substituted channels.

### Relative contributions of putative drug-binding residues

**Figure 5:**
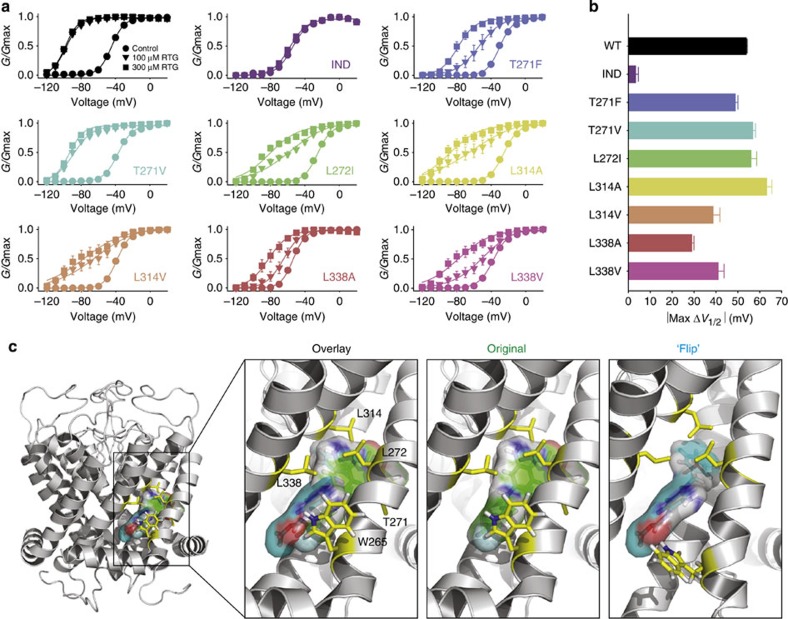
Detailed characterization of secondary retigabine binding residues and alternative binding site orientations. (**a**) Conductance–voltage relationships were gathered for the indicated KCNQ3* mutant channels (*n*=4–6 per mutant), in 0, 100 or 300 μM retigabine. (**b**) Maximal Δ*V*_1/2_ in 300 μM retigabine measured in each mutant channel. Error bars in **a**,**b** represent s.e.m. (**c**) Retigabine was docked into a molecular model of the pore-forming domain of KCNQ3 (see ref. 19). Two orientations are shown with the carbamate group in either the vicinity of Leu314 (‘original’ model) or Trp265 (‘flip’ model). The two binding models are superimposed in the ‘overlay’, showing the similar space occupied by both drug orientations.

Given the dramatic effects of the Trp265Ind substitution, we sought to clarify the contributions of other residues reported to play a role in retigabine binding. Using a chimeric approach between KCNQ1 and KCNQ3, previous work identified KCNQ3 residues Thr271 (S5 helix), Leu272 (S5 helix), Leu314 (pore helix) and Leu338 (S6 helix) as important contributors to a putative retigabine binding pocket^19^. We generated several mutations at each of these positions in KCNQ3* and tested retigabine effects over a broad concentration range (Fig. 5a,b). We observed that all functional mutants at these positions retained a large retigabine-mediated gating shift (Fig. 5b), although reduced potency was reflected by a shift to higher drug concentrations in many cases. Moreover, many of these mutations caused intrinsic shifts in channel gating in the absence of retigabine (Fig. 5a). Previous reports have suggested that many of these mutations strongly diminish retigabine action; however, our application of higher concentrations illustrates that, while potency of the drug is weakened, large gating shifts still occur. These data indicate that Thr271, Leu272, Leu314 and L338 make measurable contributions to retigabine binding affinity. However, the Trp265Ind substitution abolishes any response to retigabine at experimentally achievable concentrations. Thus, Trp265Ind remains the most structurally subtle, yet most disruptive, mutation we have identified in terms of retigabine sensitivity, highlighting the potential importance of H-bonding with the Trp265 side chain.

We next used a previously published molecular model of the retigabine binding site to investigate alternative binding modes of the drug^19^. In the published model (‘original’ in Fig. 5c) there are no obvious H-bond acceptors in the vicinity of Trp265. There are also no apparent H-bond interactions with Thr271. However, this putative binding pocket can accommodate altered orientations (for example, ‘flip’ in Fig. 5c) in which the drug occupies a similar space (see ‘overlay’ panel illustrating both binding models in Fig. 5c), but orients the carbamate moiety of retigabine in close proximity to Trp 265. A rotameric shift of Trp265 enables a close interaction between the indole N–H and a carbamate oxygen atom of retigabine. Using molecular dynamics simulations (of 12 retigabine binding sites starting in a ‘flip’ orientation, for 10 ns each), we measured the mean distance between the retigabine carbamate oxygen and indole N–H group at the end of the simulations as 4.0±0.5 Å (±indicates s.e., four binding sites were measured between 2 and 3 Å, three sites between 3 and 4 Å and five sites were >5 Å), consistent with the possible formation of a hydrogen bond in this region.

### Identification of a likely H-bond donor in retigabine

**Figure 6:**
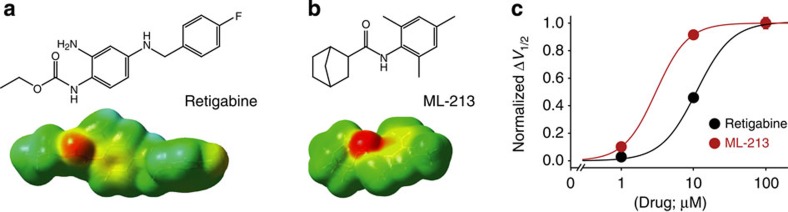
ML-213 exhibits a stronger electrostatic surface potential and higher potency than retigabine for KCNQ3* activation. (**a**,**b**) Chemical structures and electrostatic surface potentials for retigabine and ML-213. Note the increased negative surface potential in the vicinity of the carbonyl oxygen atom in ML-213. The scale for electrostatic surface potential representation is red: −80 kcal, yellow: 0 kcal, blue: +80 kcal. (**c**) Concentration–response curves for retigabine and ML-213 (*n*=5) effects on KCNQ3* channels (*n*=5). Error bars represent s.e.m.

We further investigated the mechanism of H-bonding with Trp265 by seeking potential H-bond acceptors in the retigabine molecule, a task facilitated by the availability of numerous retigabine analogues. We first considered the analogue ML-213 because it has a simplified chemical scaffold compared with retigabine and a reduced number of possible H-bond acceptors (Fig. 6a,b). We found that ML-213 is a more potent activator of KCNQ3* compared with retigabine (EC_50_=3.6±0.2 μM, *n*=5, versus 11.6±0.4 μM, *n*=5, respectively, Fig. 6c, ± indicates s.e.). We have also included the calculated electrostatic surface potentials for retigabine and ML-213, illustrating that the negative surface potential around the carbonyl oxygen is more pronounced for ML-213 (red protrusion, Fig. 6a,b).

**Figure 7:**
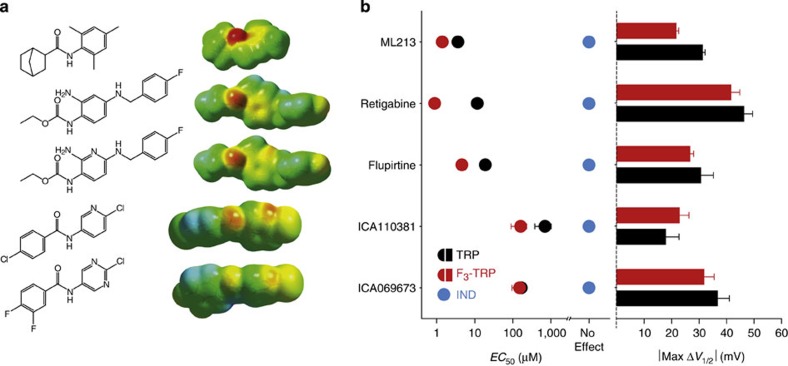
Effects of retigabine analogues correlate with electrostatic surface potential. (**a**) Chemical structures and electrostatic surface potentials for a series of retigabine analogues. All structures and surface potential maps have been aligned on the basis of the location of the conserved amide-ester bond—note the gradient of the intensity of the negative surface potential around the carbonyl oxygen atom (scaling is the same as in Fig. 6). (**b**) Summary illustrating the EC50 of each drug on Trp-rescued, F_3_-Trp-rescued and Ind-rescued KCNQ3*[Trp265TAG] channels (*n*=4–9 per data point), and the maximal efficacy (*ΔV*_1/2_) of each drug in F_3_-Trp and Trp-rescued channels (effects on Ind-rescued channels are minimal and thus have been omitted). Error bars represent s.e.m.

Expanding on these observations, we examined a spectrum of KCNQ channel activators with varying potency in KCNQ3* channels (Fig. 7). We have depicted all of the drugs tested and colorimetric representations of their calculated electrostatic surface potentials, along with the observed EC_50_ for the shift of activation *V*_1/2_ of KCNQ3*[Trp265TAG] channels (rescued with Trp, F_3_-Trp or Ind). For all drugs tested, effects were abolished by the Trp265Ind substitution, indicating a common mechanism via H-bond formation with Trp265. In addition, F_3_-Trp substitution generally resulted in higher drug potency, although this effect was not as pronounced with the less polar analogues ICA-110381 and ICA-069673. Importantly, all of the activator compounds contain a carbonyl oxygen (either in the carbamate moiety of retigabine and flupirtine, or in the amide linker in ML-213 or the ICA compounds), and it is noteworthy that the strength of the negative surface potential correlates well with the potency of the drug for KCNQ3* activation—drugs with a weaker electrostatic surface potential trend towards weaker potency (Fig. 7). The ICA compounds are very weakly potent activators of KCNQ3* channels within our typical experimental concentration range (up to 300 μM), and notably ICA-110381 caused considerably more activation in F_3_-Trp-substituted channels, demonstrating that effects of an otherwise weak channel activator can be strengthened by targeting the chemical properties of Trp265. Taken together, these findings illustrate the importance of the H-bonding propensity of Trp265, likely involved in the formation of a H-bond with a carbonyl oxygen present in retigabine and its analogues.

## Discussion

Retigabine is currently the only drug widely approved for clinical use that acts via activation of voltage-gated potassium channels^35^. Detailed investigation of this drug prototype may deepen our understanding of how drugs can act as ion channel activators, and accelerate the development of potassium channel activators with increased potency or specificity. Such drugs may have untapped therapeutic potential for management of a variety of ‘hyperexcitability’ disorders in addition to epilepsy, such as painful syndromes, hypertension and cardiac arrhythmias^9^,^36^. In addition, recent reports have highlighted retigabine effectiveness in treatment of tinnitus, Parkinson’s disease and Huntington’s disease^37^,^38^,^39^.

At the outset of our study, chimeric studies with the retigabine-insensitive KCNQ1 channel had identified the importance of a Trp residue (KCNQ3 Trp265) in the pore-forming region, near the presumed cytoplasmic channel gate. Retigabine interactions with this site had been rationalized as generic hydrophobic interactions with this conserved Trp residue (also present in KCNQ2, 4 and 5)^19^,^20^.

Although most often considered to be hydrophobic, Trp side chains can deploy diverse chemical forces in ligand interactions^40^, and these require specialized chemical-scale approaches to be distinguished^29^. There is growing recognition of the general importance of electrostatic cation–pi interactions with all aromatic side chains^29^,^41^. In addition, both Trp and Tyr side chains can act as H-bond donors. Unnatural amino-acid mutagenesis approaches allow us to parse out atomistic modes of interaction, and also hopefully mitigate the possibility of gross structural perturbations that might arise using conventional mutagenesis. By engineering KCNQ3 channels to include subtle Trp variants at position 265, our study identifies the very atoms required for retigabine effects and, further, the chemical forces involved in forming a direct interaction with retigabine. Surprisingly, our findings indicate that the critical property of Trp265 for retigabine activation, rather than hydrophobicity, is the ability to H-bond. Repositioning of the indole nitrogen atom to remove Trp H-bonding abolished the effects of retigabine and all analogues tested (Figs 3 and 7). Moreover, a Trp analogue (F_3_-Trp) with altered electrostatics but intact/enhanced H-bonding ability enhances retigabine potency (Figs 4 and 7). A valuable future direction for these experiments will be to develop an independent measure of drug binding, to distinguish whether the H-bond interaction (disrupted by the Trp265Ind mutation) is essential for drug binding, or couples drug binding to a later step involved in channel potentiation. Nevertheless, these observations highlight a rarely investigated mode of drug interactions with Trp, as Trp residues in a binding pocket are often presumed to indicate a hydrophobic site. Our findings illustrate the importance of detailed investigation of the underlying chemistry of these binding sites to better understand mechanisms of drug action.

**Figure 8:**
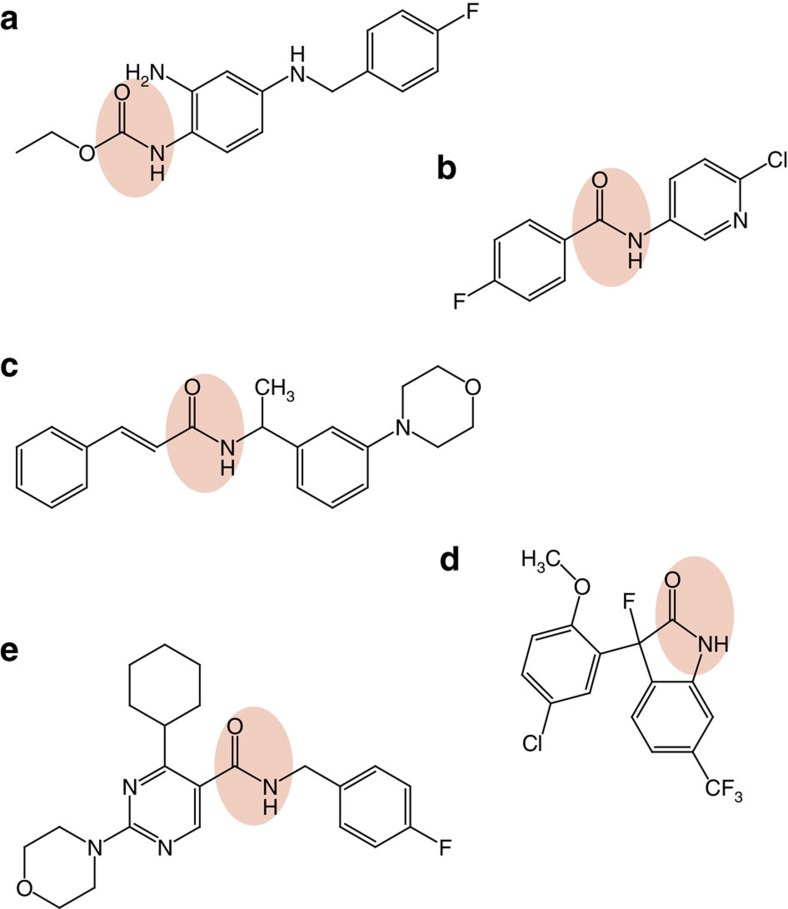
Diverse structures of KCNQ openers. Multiple structures of KCNQ channel openers are presented to highlight the overall features of an amide group flanked by various ring structures. Our findings highlight the importance of the amide carbonyl for interaction with KCNQ3 Trp 265 and likely equivalent positions in KCNQ2, 4 and 5. Drugs depicted are (**a**) retigabine, (**b**) ztz-240 (described in ref. 24), (**c**) acrylamide (s)-1, (**d**) BMS-204352 and (**e**) an unnamed experimental drug described in ref. 43.

By demonstrating the effects of H-bond strength on a range of KCNQ3 activators, our findings suggest the importance of a carbonyl oxygen (usually in a carbamate or amide moiety) for the formation of a negative electrostatic surface potential to act as a H-bond acceptor. Previous screens of compound libraries have investigated important physicochemical features important for drug activity on KCNQ2–5 channels. Several studies have reported the importance of an amide bond (or a carbonyl oxygen) as an essential element of the pharmacophore, although the relationship between this functional group and the H-bonding propensity of Trp265 has not been recognized^9^,^42^,^43^,^44^. Notably, KCNQ activators with marked structural diversity appear to act through Trp265 (several examples are shown in Fig. 8), and these share the common feature of a carbonyl oxygen that we suggest acts as an essential hydrogen bond acceptor. Supporting this notion, our findings illustrate that ML-213 is an effective KCNQ3 activator, correlated with its strong surface potential relative to other drugs tested. In the context of understanding drug interactions with ion channels and other receptors, these observations illustrate the importance of investigating specific chemical forces that enable drug interactions with aromatic side chains known to contribute to binding sites in other ion channels (for example, voltage-gated Na^+^ channels, human ether-a-go-go related gene product (hERG))^45^,^46^,^47^. These insights could guide rational ‘tuning’ of the properties of certain functional groups to alter drug properties in desirable ways.

On the basis of the magnitude of the retigabine-induced shift of the conductance–voltage relationship, saturating effects of retigabine are estimated to stabilize channel opening by ∼6 kcal mol^−1^ (calculated as Δ*G=*Δ(*zFV*_1/2_)). As a comparison, formation of a H-bond between a carbonyl and amino groups (N-H: O=C) is generally quite weak, on the order of 2 kcal mol^−1^. A tetrameric KCNQ channel likely contains four potential retigabine binding sites, and our characterization of heteromeric channels with fewer binding sites (Fig. 1c) indicates that multiple sites may be occupied in saturating retigabine concentrations. Thus, the energetics of the full retigabine-mediated gating shift can be reasonably attributed to multiple retigabine molecules interacting with Trp265 via a H-bond. Although not as prominent as the Trp265 indole nitrogen, other residues also make additional measurable contributions to the overall energy of retigabine binding (Fig. 5 and ref. 19). It should also be noted that cooperativity between retigabine binding sites has not been investigated in detail, and so it is not known how multiple retigabine binding events may interact and influence channel function. Investigation of libraries of retigabine analogues has highlighted that variable aromatic ring substituents can alter the apparent affinity, maximal effect and subunit specificity^24^,^43^. For example, many ring substituents (such as halogenation) alter the polarity of the carbonyl oxygen and thereby alter drug interactions with Trp265. In addition, it seems likely that diverse flanking structures (potentially by interacting with the auxiliary retigabine binding residues investigated in Fig. 5) could influence the orientation of the carbonyl oxygen relative to Trp265.

A final noteworthy detail is that, although our findings delineate an atomic basis for the retigabine interaction with KCNQ3 Trp265 (and analogous residues in KCNQ2,4,5), there is significant evidence for additional interaction sites that enable potentiation of certain KCNQ subunits. For example, the ICA compounds tested (and other related compounds such as ztz-240), along with a family of diclofenac derivatives, have been reported to influence KCNQ2 channels independent of the conserved S5 Trp residue and exhibit dramatically stronger effects in KCNQ2 relative to KCNQ3 (refs 22, 24, 25, 30). In addition, unrelated KCNQ openers such as zinc pyrithione do not appear to depend on the hydrogen bond interaction that we have identified in this study^48^. Therefore, ongoing examination of drug binding to different KCNQ channel subtypes may help to reveal additional sites for KCNQ potentiation.

In conclusion, we have used unnatural amino-acid mutagenesis and available retigabine analogues to localize the effects of retigabine binding to a single H-bond interaction with KCNQ3 Trp265. These findings highlight an unusual mode of drug interaction with a Trp side chain, and demonstrate the importance of careful investigation of the mechanism of drug interactions to guide rational modification of therapeutic compounds.

## Methods

### Molecular biology and *in vivo* nonsense suppression

KCNQ2 (human) and KCNQ3 (human) channel cDNAs were propagated and manipulated using the pTLN vector (gifts of Dr M. Taglialatela and Dr T. Jentsch). Introduction of point mutations was accomplished using PCR to combine two fragments each of which contained the mutation in an overlapping region of the sequence (‘two-step’ PCR method). To accomplish this, we used standard PCR approaches to amplify a 5′ fragment and a 3′ fragment (using a ‘flanking primer’ paired with a mutagenic primer). These fragments were then mixed along with the ‘flanking primer’ pair for a second round of PCR to combine the overlapping fragments. The resulting fragment was then subcloned into the appropriate parent vector using EcoRI and NotI restriction enzymes, and the sequence was verified by Sanger sequencing approaches (Genewiz). Sequences for the flanking primers and the ‘forward’ mutagenic primers are provided in Supplementary Table 1 for all mutations that were generated (‘reverse’ mutagenic primers are simply the reverse complement). In all experiments involving homomeric expression of KCNQ3 channels (including *in vivo* nonsense suppression experiments), the Ala315Thr mutation was introduced to enable efficient trafficking of homomeric KCNQ3. KCNQ3 does not efficiently form functional channels; however, the Ala315Thr mutation enables efficient KCNQ3 functional expression without co-injection of KCNQ2 mRNA (throughout the text we refer to KCNQ3[Ala315Thr] as KCNQ3*)^49^. The plasmid encoding CivSP was kindly provided by Dr Y. Okamura. Complementary RNA was transcribed from the cDNA using the mMessage mMachine Kit (Ambion). Stage V–VI *Xenopus laevis* oocytes were prepared as previously described and were injected with cRNA alone or cRNA plus synthetic tRNA (for nonsense suppression, described below). Oocyte preparation was carried out using a protocol approved by the University of British Columbia Animal Care Committee, in accordance with the Canadian Council for Animal Care guidelines. We used female *Xenopus laevis* frogs 100 g or greater in size. After injection, oocytes were incubated for 12–48 h at 18 °C before recording.

Fluorinated Trp derivatives (5-F_1_-Trp; 7-F_1_-Trp; 5,7-F_2_-Trp (F_2_-Trp) and 5,6,7-F_3_-Trp (F_3_-Trp) were purchased from Asis Chem (Watertown, MA) and Sigma-Aldrich (St Louis, MO). The ‘Ind’ Trp variant was synthesized in-house as described previously^31^,^50^. Incorporation of unnatural amino acids into ion channel proteins was carried out as described extensively in prior publications^29^,^34^,^51^. Briefly, unnatural amino acids (aa) were protected with nitroveratryloxycarbonyl and were activated as the cyanomethyl ester, which were then coupled to the dinucleotide pdCpA (GE Healthcare/Dharmacon, Lafayette, CO). This aminoacyl dinucleotide was subsequently ligated to a modified (G73) *Tetrahymena thermophile* tRNA. The amino-acylated tRNA-aa was deprotected by ultraviolet irradiation immediately before oocyte injection. In a typical experiment, 80 ng of tRNA-aa and 40 ng of KCNQ3 cRNA were injected in a 50-nl volume. In control experiments, the cRNA alone or the cRNA together with a tRNA coupled to pdCpA but without an appended amino acid were injected (these parallel control experiments were conducted every experimental day and the results are described throughout the text).

### Two-electrode voltage clamp recordings

Voltage-clamped potassium currents were recorded in standard Ringers solution (in mM): 116 NaCl, 2 KCl, 1 MgCl_2_, 0.5 CaCl_2_, 5 HEPES (pH 7.4) using an OC-725C voltage clamp (Warner, Hamden, CT). Glass microelectrodes were backfilled with 3 M KCl and had resistances of 0.1–1 MΩ. Data were filtered at 5 kHz and digitized at 10 kHz using a Digidata 1440A (Molecular Devices) controlled by the pClamp 10 software (Molecular Devices). Drugs were purchased from Toronto Research Chemicals (retigabine) or Tocris (ML-213, flupirtine, ICA-069673, ICA-110381), stored as 100 mM stocks in dimethylsulphoxide and diluted to working concentrations each experimental day.

### Molecular simulations of retigabine binding

A published model of retigabine docked to the KCNQ3 pore domain was used as the starting point of simulations^19^. In this model the retigabine molecule is positioned with its potential H-bond donor carbonyl oxygen pointed away from Trp265. Therefore, the retigabine was rotated by ∼180° (‘flipped’ upside down). Three further retigabine molecules were docked into a similar position resulting in a model with all four binding sites occupied. This model was energy-minimized using force field AMBER03 and the constraints for the retigabine molecules were assigned with semi-empirical quantum mechanics (QM) using force field NOVA. Subsequently, a short molecular dynamics (MD) simulation was run while constraining the distance between the Trp265 N–H group and the carbonyl oxygen of retigabine to a distance of 4±1.5 Å. The resulting model contained four retigabine molecules in the ‘flip’ position, and all four Trp265 side chains in a rotated position (see Fig. 5c). Starting with this model, zero, one, two (in opposing or adjacent subunits), three or four retigabines were deleted from the respective model generating all possible combinations of occupied binding-site configurations. Each resulting model was inserted into a virtual membrane and MD simulations were run as described previously^52^. In brief, models were incorporated into membranes, the simulation box was filled with 0.9 mM NaCl/H_2_O and, subsequently, all-atom-mobile simulations were performed for 10 ns to reach stable conformations using YASARA Structure version 10.1 (AMBER03, 2 fs time steps; YASARA Biosciences GmbH, Vienna, Austria). The average structures of all models were calculated and the distance between the Trp265 N-H group and the carbonyl oxygen of retigabine was measured individually in each occupied binding site.

### Data analysis

Voltage dependence of channel activation was fitted with a standard single component Boltzmann equation of the form *G/G*max=1/(1+*e*^*−(V−V*_1/2_^)/*k* where *V*_1/2_ is the voltage where channels exhibit half-maximal activation, and *k* is a slope factor reflecting the voltage range over which an *e*-fold change in Po is observed. For generation of concentration–response curves to retigabine and derivatives, Δ*V*_1/2_ (the shift in half-activation voltage) was normalized to the maximal Δ*V*_1/2_ observed, and fit with the following equation: normalized Δ*V*_1/2_=1/(1+EC_50_/[drug]). Statistical tests and significance are described in figure legends throughout the text.

## Additional information

**How to cite this article:** Kim, R. Y. *et al*. Atomic basis for therapeutic activation of neuronal potassium channels. *Nat. Commun*. 6:8116 doi: 10.1038/ncomms9116 (2015).

## Supplementary Material

Supplementary Information Supplementary Table 1

## Figures and Tables

**Table 1:** Activation gating parameters for unnatural amino-acid substitutions of KCNQ3*[Trp265TAG] channels.

Substitution	*V*_1/2_ (mV±s.e.m.)	Slope factor (mV±s.e.m.)	*n*
WT-Trp	−44±1	7.5±0.5	5
265TAG-Trp	−43±2	7.9±0.5	12
265TAG-F_1_-Trp	−38±2	9.3±0.5	9
265TAG-F_2_-Trp	−48±2	9.9±0.8	7
265TAG-F_3_-Trp	−62.3±3	10.9±0.8	12
265TAG-Ind	−48±2	7.3±0.6	7

Fitting of conductance–voltage relationships (see Methods) yielded *V*_1/2_ (voltage where activation is half-maximal) and the slope factor *k* (voltage range where fractional activation changes *e*-fold). Data are presented as mean±s.e.m.

## References

[b1] Brodie M. J. Antiepileptic drug therapy the story so far. Seizure 19, 650–655 (2010). 21075011 10.1016/j.seizure.2010.10.027

[b2] Kwan P. . Definition of drug resistant epilepsy: consensus proposal by the *ad hoc* Task Force of the ILAE Commission on Therapeutic Strategies. Epilepsia 51, 1069–1077 (2010). 19889013 10.1111/j.1528-1167.2009.02397.x

[b3] Pati S. & Alexopoulos A. V. Pharmacoresistant epilepsy: from pathogenesis to current and emerging therapies. Cleve. Clin. J. Med. 77, 457–467 (2010). 20601619 10.3949/ccjm.77a.09061

[b4] Lutas A. & Yellen G. The ketogenic diet: metabolic influences on brain excitability and epilepsy. Trends Neurosci. 36, 32–40 (2013). 23228828 10.1016/j.tins.2012.11.005 PMC3534786

[b5] Yellen G. Ketone bodies, glycolysis, and KATP channels in the mechanism of the ketogenic diet. Epilepsia 49, (Suppl 8): 80–82 (2008). 10.1111/j.1528-1167.2008.01843.x PMC2646251 19049596

[b6] Klassen T. . Exome sequencing of ion channel genes reveals complex profiles confounding personal risk assessment in epilepsy. Cell 145, 1036–1048 (2011). 21703448 10.1016/j.cell.2011.05.025 PMC3131217

[b7] Luszczki J. J. Third-generation antiepileptic drugs: mechanisms of action, pharmacokinetics and interactions. Pharmacol. Rep. 61, 197–216 (2009). 19443931 10.1016/s1734-1140(09)70024-6

[b8] Wilcox K. S. . Issues related to development of new antiseizure treatments. Epilepsia 54, (Suppl 4): 24–34 (2013). 23909851 10.1111/epi.12296 PMC3947404

[b9] Miceli F., Soldovieri M. V., Martire M. & Taglialatela M. Molecular pharmacology and therapeutic potential of neuronal Kv7-modulating drugs. Curr. Opin. Pharmacol. 8, 65–74 (2008). 18061539 10.1016/j.coph.2007.10.003

[b10] Brodie M. J. . Efficacy and safety of adjunctive ezogabine (retigabine) in refractory partial epilepsy. Neurology 75, 1817–1824 (2010). 20944074 10.1212/WNL.0b013e3181fd6170

[b11] Orhan G., Wuttke T. V., Nies A. T., Schwab M. & Lerche H. Retigabine/Ezogabine, a KCNQ/K(V)7 channel opener: pharmacological and clinical data. Expert. Opin. Pharmacother. 13, 1807–1816 (2012). 22783830 10.1517/14656566.2012.706278

[b12] Blackburn-Munro G., Dalby-Brown W., Mirza N. R., Mikkelsen J. D. & Blackburn-Munro R. E. Retigabine: chemical synthesis to clinical application. CNS Drug Rev. 11, 1–20 (2005). 15867950 10.1111/j.1527-3458.2005.tb00033.x PMC6741764

[b13] Porter R. J., Nohria V. & Rundfeldt C. Retigabine. Neurotherapeutics 4, 149–154 (2007). 17199031 10.1016/j.nurt.2006.11.012 PMC7479689

[b14] Tatulian L., Delmas P., Abogadie F. C. & Brown D. A. Activation of expressed KCNQ potassium currents and native neuronal M-type potassium currents by the anti-convulsant drug retigabine. J. Neurosci. 21, 5535–5545 (2001). 11466425 10.1523/JNEUROSCI.21-15-05535.2001 PMC6762632

[b15] Otto J. F., Kimball M. M. & Wilcox K. S. Effects of the anticonvulsant retigabine on cultured cortical neurons: changes in electroresponsive properties and synaptic transmission. Mol. Pharmacol. 61, 921–927 (2002). 11901232 10.1124/mol.61.4.921

[b16] Shah M., Mistry M., Marsh S. J., Brown D. A. & Delmas P. Molecular correlates of the M-current in cultured rat hippocampal neurons. J. Physiol. 544, 29–37 (2002). 12356878 10.1113/jphysiol.2002.028571 PMC2290582

[b17] Wickenden A. D., Yu W., Zou A., Jegla T. & Wagoner P. K. Retigabine, a novel anti-convulsant, enhances activation of KCNQ2/Q3 potassium channels. Mol. Pharmacol. 58, 591–600 (2000). 10953053 10.1124/mol.58.3.591

[b18] Main M. J. . Modulation of KCNQ2/3 potassium channels by the novel anticonvulsant retigabine. Mol. Pharmacol. 58, 253–262 (2000). 10908292 10.1124/mol.58.2.253

[b19] Lange W. . Refinement of the binding site and mode of action of the anticonvulsant Retigabine on KCNQ K+ channels. Mol. Pharmacol. 75, 272–280 (2009). 19015229 10.1124/mol.108.052282

[b20] Schenzer A. . Molecular determinants of KCNQ (Kv7) K+ channel sensitivity to the anticonvulsant retigabine. J. Neurosci. 25, 5051–5060 (2005). 15901787 10.1523/JNEUROSCI.0128-05.2005 PMC6724866

[b21] Xiong Q., Gao Z., Wang W. & Li M. Activation of Kv7 (KCNQ) voltage-gated potassium channels by synthetic compounds. Trends Pharmacol. Sci. 29, 99–107 (2008). 18206251 10.1016/j.tips.2007.11.010

[b22] Boehlen A. . The new KCNQ2 activator 4-Chlor-N-(6-chlor-pyridin-3-yl)-benzamid displays anticonvulsant potential. Br. J. Pharmacol. 168, 1182–1200 (2013). 23176257 10.1111/bph.12065 PMC3594676

[b23] Yu H. . Discovery, synthesis, and structure activity relationship of a series of N-Aryl- bicyclo[2.2.1]heptane-2-carboxamides: characterization of ML213 as a novel KCNQ2 and KCNQ4 potassium channel opener. ACS Chem. Neurosci. 2, 572–577 (2011). 22125664 10.1021/cn200065b PMC3223964

[b24] Gao Z. . Isoform-specific prolongation of Kv7 (KCNQ) potassium channel opening mediated by new molecular determinants for drug-channel interactions. J. Biol. Chem. 285, 28322–28332 (2010). 20584905 10.1074/jbc.M110.116392 PMC2934696

[b25] Peretz A. . A tale of switched functions: from cyclooxygenase inhibition to M-channel modulation in new diphenylamine derivatives. PLoS ONE 2, e1332 (2007). 18159230 10.1371/journal.pone.0001332 PMC2131780

[b26] Wickenden A. D., Zou A., Wagoner P. K. & Jegla T. Characterization of KCNQ5/Q3 potassium channels expressed in mammalian cells. Br. J. Pharmacol. 132, 381–384 (2001). 11159685 10.1038/sj.bjp.0703861 PMC1572592

[b27] Murata Y., Iwasaki H., Sasaki M., Inaba K. & Okamura Y. Phosphoinositide phosphatase activity coupled to an intrinsic voltage sensor. Nature 435, 1239–1243 (2005). 15902207 10.1038/nature03650

[b28] Nowak M. W. . *In vivo* incorporation of unnatural amino acids into ion channels in Xenopus oocyte expression system. Methods Enzymol. 293, 504–529 (1998). 9711626 10.1016/s0076-6879(98)93031-2

[b29] Beene D. L., Dougherty D. A. & Lester H. A. Unnatural amino acid mutagenesis in mapping ion channel function. Curr. Opin. Neurobiol. 13, 264–270 (2003). 12850209 10.1016/s0959-4388(03)00068-0

[b30] Peretz A. . Targeting the voltage sensor of Kv7.2 voltage-gated K+ channels with a new gating-modifier. Proc. Natl Acad. Sci. USA 107, 15637–15642 (2010). 20713704 10.1073/pnas.0911294107 PMC2932606

[b31] Lacroix J. J. . Intermediate state trapping of a voltage sensor. J. Gen. Physiol. 140, 635–652 (2012). 23183699 10.1085/jgp.201210827 PMC3514728

[b32] Dougherty D. A. Cation-pi interactions in chemistry and biology: a new view of benzene, Phe, Tyr, and Trp. Science 271, 163–168 (1996). 8539615 10.1126/science.271.5246.163

[b33] Deutsch C. J. & Taylor J. S. Intracellular pH as measured by 19F NMR. Ann. N Y Acad. Sci. 508, 33–47 (1987). 3501935 10.1111/j.1749-6632.1987.tb32892.x

[b34] Pless S. A., Galpin J. D., Niciforovic A. P., Kurata H. T. & Ahern C. A. Hydrogen bonds as molecular timers for slow inactivation in voltage-gated potassium channels. Elife 2, e01289 (2013). 24327560 10.7554/eLife.01289 PMC3852034

[b35] Martyn-St J. M. . The efficacy and safety of retigabine and other adjunctive treatments for refractory partial epilepsy: a systematic review and indirect comparison. Seizure 21, 665–678 (2012). 22902288 10.1016/j.seizure.2012.07.011

[b36] Jepps T. A., Olesen S. P. & Greenwood I. A. One man's side effect is another man's therapeutic opportunity: targeting Kv7 channels in smooth muscle disorders. Br. J. Pharmacol. 168, 19–27 (2013). 22880633 10.1111/j.1476-5381.2012.02133.x PMC3569999

[b37] Li S., Choi V. & Tzounopoulos T. Pathogenic plasticity of Kv7.2/3 channel activity is essential for the induction of tinnitus. Proc. Natl Acad. Sci. USA 110, 9980–9985 (2013). 23716673 10.1073/pnas.1302770110 PMC3683764

[b38] Sander S. E., Lemm C., Lange N., Hamann M. & Richter A. Retigabine, a K(V)7 (KCNQ) potassium channel opener, attenuates L-DOPA-induced dyskinesias in 6-OHDA-lesioned rats. Neuropharmacology 62, 1052–1061 (2012). 22079161 10.1016/j.neuropharm.2011.10.016

[b39] Cao Y. . Rescue of homeostatic regulation of striatal excitability and locomotor activity in a mouse model of Huntington's disease. Proc. Natl Acad. Sci. USA 112, 2239–2244 (2015). 25646456 10.1073/pnas.1405748112 PMC4343133

[b40] Burley S. K. & Petsko G. A. Aromatic-aromatic interaction: a mechanism of protein structure stabilization. Science 229, 23–28 (1985). 3892686 10.1126/science.3892686

[b41] Gallivan J. P. & Dougherty D. A. Cation-pi interactions in structural biology. Proc. Natl Acad. Sci. USA 96, 9459–9464 (1999). 10449714 10.1073/pnas.96.17.9459 PMC22230

[b42] Wu Y. J. . Synthesis and structure-activity relationship of acrylamides as KCNQ2 potassium channel openers. J Med. Chem. 47, 2887–2896 (2004). 15139767 10.1021/jm0305826

[b43] Wu Y. J. & Dworetzky S. I. Recent developments on KCNQ potassium channel openers. Curr. Med. Chem. 12, 453–460 (2005). 15720253 10.2174/0929867053363045

[b44] Bentzen B. H. . The acrylamide (S)-1 differentially affects Kv7 (KCNQ) potassium channels. Neuropharmacology 51, 1068–1077 (2006). 16904708 10.1016/j.neuropharm.2006.07.001

[b45] Sanguinetti M. C. . Physicochemical basis for binding and voltage-dependent block of hERG channels by structurally diverse drugs. Novartis. Found. Symp. 266, 159–166 (2005). 16050267

[b46] Mitcheson J. S., Chen J., Lin M., Culberson C. & Sanguinetti M. C. A structural basis for drug-induced long QT syndrome. Proc. Natl Acad. Sci. USA 97, 12329–12333 (2000). 11005845 10.1073/pnas.210244497 PMC17341

[b47] Pless S. A., Galpin J. D., Frankel A. & Ahern C. A. Molecular basis for class Ib anti-arrhythmic inhibition of cardiac sodium channels. Nat. Commun. 2, 351 (2011). 21673672 10.1038/ncomms1351

[b48] Xiong Q., Sun H. & Li M. Zinc pyrithione-mediated activation of voltage-gated KCNQ potassium channels rescues epileptogenic mutants. Nat. Chem. Biol. 3, 287–296 (2007). 17435769 10.1038/nchembio874

[b49] Zaika O., Hernandez C. C., Bal M., Tolstykh G. P. & Shapiro M. S. Determinants within the turret and pore-loop domains of KCNQ3 K+ channels governing functional activity. Biophys. J. 95, 5121–5137 (2008). 18790849 10.1529/biophysj.108.137604 PMC2586577

[b50] Belokon Y. N. . Nucleophilic addition to an achiral dehydroalanine Schiff base Ni(II) complex as a route to amino acids. A case of stereodetermining asymmetric protonation in the presence of TADDOL. ARKIVOC. 3, 132–150 (2004).

[b51] Pless S. A. . A novel mechanism for fine-tuning open-state stability in a voltage-gated potassium channel. Nat. Commun. 4, 1784 (2013). 23653196 10.1038/ncomms2761 PMC3644096

[b52] Strutz-Seebohm N. . Structural basis of slow activation gating in the cardiac I Ks channel complex. Cell. Physiol. Biochem. 27, 443–452 (2011). 21691061 10.1159/000329965

