# Supplementary material for: Atomic basis for therapeutic activation of neuronal potassium channels
Source: Nat Commun. 2015 Sep 3;6:8116. doi: 10.1038/ncomms9116 (PMC4561856; doi:10.1038/ncomms9116)
Supplement: Supplementary Information — Supplementary Table 1 [file ncomms9116-s1.pdf]

Supplementary Table 1

Primers for mutagenesis of KCNQ2 and KCNQ3 channels:

Q2 mutagenesis primers:

Forward flanking	5’-CCAAGCGCAACGCCTTCTACC-3’
Reverse flanking	5’- GGCTGGTTTAGTGGTAACCAG-3’
KCNQ2 Trp236Phe	5’-GCTGGTCACTGCC**TTC**TACATCGGCTTCC-3’

Q3 mutagenesis primers:

Forward flanking	5’- GGCGACGTGGAGCAAGTCACC-3’
Reverse flanking	5’-TAAGGCCTCAAAGTCTCCTTG-3’
KCNQ3 Trp265Phe	5’-GCAAAGAACTCATCACGGCC**TTC**TACATCGGTTTCCTGAC-3’
KCNQ3 Trp265Leu	5’-GCAAAGAACTCATCACGGCC**TTG**TACATCGGTTTCCTGAC-3’
KCNQ3 Trp265Ser	5’-GCAAAGAACTCATCACGGCC**TCG**TACATCGGTTTCCTGAC-3’
KCNQ3 Trp265Thr	5’-GCAAAGAACTCATCACGGCC**ACG**TACATCGGTTTCCTGAC-3’
KCNQ3 Trp265Tyr	5’-GCAAAGAACTCATCACGGCC**TAC**TACATCGGTTTCCTGAC-3’
KCNQ3 Trp265TAG	5’-GCAAAGAACTCATCACGGCC**TAG**TACATCGGTTTCCTGAC-3’
KCNQ3 Thr271Phe	5’-GGTACATCGGTTTCCTG**TTT**CTCATCCTTTCTTCATTTC-3’
KCNQ3 Thr271Val	5’-GGTACATCGGTTTCCTG**GTA**CTCATCCTTTCTTCATTTC-3’
KCNQ3 Leu272Ile	5’-GGTACATCGGTTTCCTGACA**ATC**ATCCTTTCTTCATTTC-3’
KCNQ3 Leu314Ala	5’-GGGGCCTGATCACAG**CGG**CCACCATTGGCTATGG-3’
KCNQ3 Leu314Val	5’-GGGGCCTGATCACAG**TGG**CCACCATTGGCTATGG-3’
KCNQ3 Leu338Ala	5’-GCCGCCACCTTTTCCG**CAA**TTGGCGTCTCCTTTTTTGCC-3’
KCNQ3 Leu338Val	5’-GCCGCCACCTTTTCCG**TAA**TTGGCGTCTCCTTTTTTGCC-3’
